# Spontaneous ruptured congenital bronchial diverticulum presenting with total lung collapse and chronic empyema thoracic

**DOI:** 10.1093/icvts/ivaf060

**Published:** 2025-03-26

**Authors:** Padungkiat Tangpiroontham

**Affiliations:** Department of Cardiothoracic Surgery, Bangkok Hospital, Bangkok, Thailand

**Keywords:** bronchial diverticulum, chronic empyema thoracis, ruptured bronchial diverticulum

## Abstract

Spontaneous rupture of a congenital bronchial diverticulum resulting in total lung collapse and chronic empyema thoracis is a rare condition that presents considerable challenges in preoperative diagnosis and perioperative management. Thorough interpretation of imaging studies and bronchoscopy is crucial for effective surgical planning. This article describes a successful surgical intervention employing primary repair in conjunction with decortication.

## BACKGROUND

Tracheobronchial diverticula are rare anatomical entities that can be classified into two subtypes: (i) congenital diverticula, which consist of all layers of the tracheobronchial wall, and (ii) acquired diverticula, which are limited to the mucosal layer. Bronchial diverticula are often discovered incidentally through computed tomography (CT) scans and are more prevalent in smokers, typically located in the subcarinal area [1]. These diverticula can mimic other conditions, such as pneumomediastinum [2]. The occurrence of ruptured bronchial diverticula is extremely rare, with few reports documenting a ruptured tracheal diverticulum resulting from barotrauma [3, 4]. We present a case of successful surgical repair of a spontaneously ruptured congenital bronchial diverticulum in a patient who experienced total lung collapse and chronic empyema thoracis.

## PATIENTS AND METHODS

**Figure 1:**
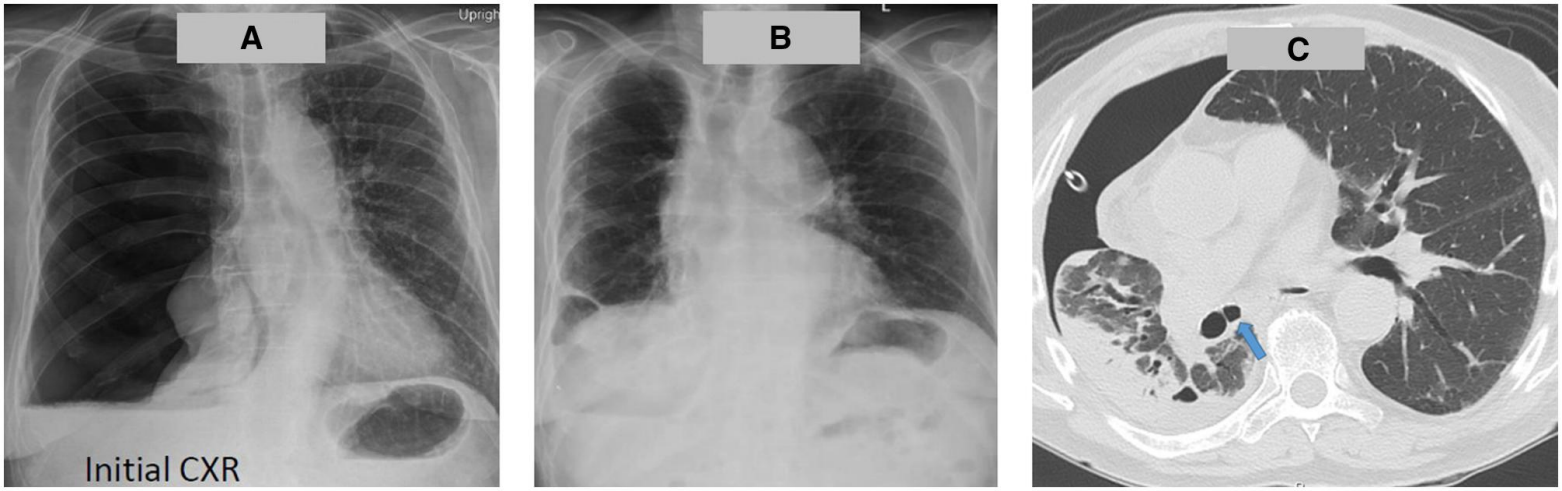
Operative finding and postoperative course: initial CXR (**A**), 3-month postoperative CXR (**B**) and bronchial diverticulum on CT chest (**C**)

A 65-year-old male patient presented to a local hospital with a 6-week history of dry cough, low-grade fever and weight loss, initially treated for bronchitis. A subsequent chest X-ray (CXR) revealed total lung collapse (Fig. 1A), leading to a preliminary diagnosis of a ruptured lung bleb. An intercostal drain was placed, which revealed an air leak and turbid yellow fluid. Follow-up CXR showed partial lung expansion. He was then referred for surgical management. Upon arrival, CT imaging of the chest indicated trapped right lower lobe, right middle lobe and residual pleural effusion. The surgical plan included fibre-optic bronchoscopy, video-assisted thoracoscopic surgery exploration, chest washout and repair of the air leak.

**Figure 2:**
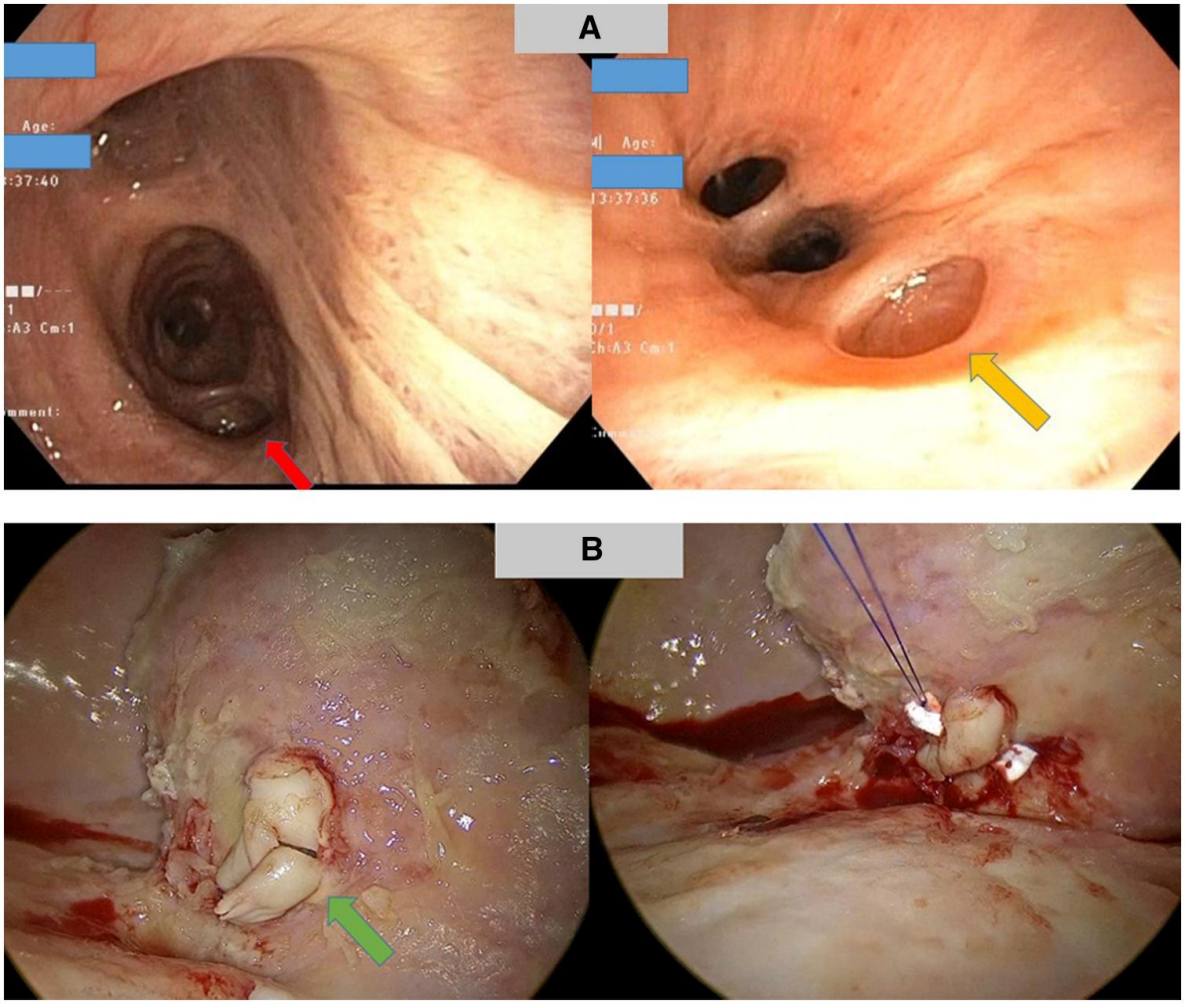
Intraoperative finding. Red arrow marked diverticulum in BI, yellow arrow marked diverticulum in RLL bronchus (**A**), green arrow marked bronchial diverticulum pre and post repair (**B**). BI: bronchus intermedius; RLL: right lower lobe

Fibre-optic bronchoscopy revealed multiple bronchial diverticula located at the bronchus intermedius, right upper lobe, and right lower lobe, varying in size from 3 to 10 mm (Fig. 2A). Uniportal video-assisted thoracoscopic surgery exploration uncovered diffusely thickened visceral and parietal pleura, with foul-smelling greenish fibrin primarily in the basilar region. Upon removal of the fibrin, a ruptured cartilaginous bronchial diverticulum was identified (Fig. 2B), along with infected material in the diverticulum adjacent to the right lower pulmonary vein, presenting a significant air leak during right lung ventilation. A hybrid minithoracotomy was performed for primary repair using three horizontal mattress sutures of Prolene 4/0 with pledget reinforcement (Fig. 2B). Decortication was subsequently carried out. No air leak was observed at the sutured site under 25 cmH2O lung reinflation. The patient had an uneventful postoperative course, with the intercostal drain removed on postoperative day 11. At 3 months post-discharge, CXR indicated full lung expansion (Fig. 1B). Retrospective analysis revealed bronchial diverticula, which were identifiable on preoperative CT imaging (Fig. 1C).

## DISCUSSION

To the best of our knowledge, this is the first case report of spontaneous ruptured congenital bronchial diverticulum combined with chronic empyema thoracis; it made the operation very challenging. The possible cause is believed to be the chronic accumulation of infected material within the diverticulum. We considered primary repair with suture, and decortication were the best option at that moment. If primary repair had failed, we would have planned to proceed with muscle flap with or without Clagett window. Shin *et al.* [5] described successful management of complicated empyema with BPF using bronchial block, Watanabe spigot and embolization under Venovenous ECMO support during respiratory failure. With increasing Low dose CT [1], tracheobronchial diverticulum may be found more frequent, risk of rupture and infection need to be explained to the patient.

## CONCLUSION

Spontaneous rupture of a congenital bronchial diverticulum, resulting in total lung collapse and chronic empyema thoracis, is a rare condition. Primary repair combined with decortication effectively controlled the air leak and led to successful lung re-expansion. Further cases are necessary to better understand optimal management strategies for similar presentations.

## Data Availability

The data underlying this article are available in the article.

## Notes

Presented as a poster presentation #79 at ESTS annual meeting 2023, 4–6 June 2023, Milan, Italy.
